# Vascularized lymph node flaps can survive on venous blood without an arterial inflow: an experimental model describing the dynamics of venous flow using indocyanine green angiography (With video)

**DOI:** 10.1093/burnst/tkad019

**Published:** 2023-07-19

**Authors:** Ke Li, Fabio Nicoli, Chunxiao Cui, Yan Wo, Ning Fei Liu, Shaoqing Feng, Wenjing Xi, Peiru Min, Yixin Zhang

**Affiliations:** Department of Plastic and Reconstructive Surgery, Shanghai Ninth People’s Hospital, Shanghai JiaoTong University School of Medicine, Shanghai 200000, China; Translational and Clinical Research Institute, Newcastle University, Newcastle upon Tyne NE1 1AD, UK; Department of Plastic and Reconstructive Surgery, University of Rome “Tor Vergata” 999016, Italy; Department of Plastic and Reconstructive Surgery, Northumbria NHS Trust, Newcastle upon Tyne NE1 1AD, UK; Department of Facial Plastic and Reconstructive Surgery, ENT Institute, Eye and ENT Hospital, Fudan University, Shanghai 200000, China; Department of Anatomy and Physiology, School of Medicine, Shanghai Jiao Tong University, Shanghai 200000, P. R. China; Department of Plastic and Reconstructive Surgery, Shanghai Ninth People’s Hospital, Shanghai JiaoTong University School of Medicine, Shanghai 200000, China; Department of Plastic and Reconstructive Surgery, Shanghai Ninth People’s Hospital, Shanghai JiaoTong University School of Medicine, Shanghai 200000, China; Department of Plastic and Reconstructive Surgery, Shanghai Ninth People’s Hospital, Shanghai JiaoTong University School of Medicine, Shanghai 200000, China; Department of Plastic and Reconstructive Surgery, Shanghai Ninth People’s Hospital, Shanghai JiaoTong University School of Medicine, Shanghai 200000, China; Department of Plastic and Reconstructive Surgery, Shanghai Ninth People’s Hospital, Shanghai JiaoTong University School of Medicine, Shanghai 200000, China

**Keywords:** Vein circulation, Venous, Lymph node, Flap, Vascularized lymph node flap, Lymphedema, Indocyanine green

## Abstract

**Background:**

Several surgeons have described studies of free-tissue transfers using veins instead of arteries. These innovative microsurgical techniques can offer several advantages, such as an easier dissection during flap harvesting, and represent an alternative during an accidental surgical mistake or development of new surgical procedures. The purpose of this study was to describe and explore different constructs of vascularized lymph node transfer (VLNT) only based on venous blood flow in a mouse model, evaluate their blood flow microcirculation through indocyanine green (ICG) angiography and investigate the lymphatic drainage function and the lymph nodes’ structures.

**Methods:**

Five types of venous lymph node flaps (LNF) were created and investigated: Types IA, IB, IC, IIA and IIB were developed by ICG intraoperatively (with videos in the article). Seven weeks later, by applying methylene blue, the recanalization of the lymphatic vessels between the LNF and the recipient site was detected. Lymph nodes were collected at the same time and their structures were analyzed by hematoxylin and eosin staining analysis.

**Results:**

All of the venous LNFs developed except Type IC. Seven weeks later, methylene blue flowed into Types IA, IB, IIA and IIB from recipient sites. When comparing with arteriovenous lymph node, the medullary sinus was diffusely distributed in venous lymph nodes. The proportion of cells was significantly reduced (*p* < 0.05). The artery diameters were significantly smaller (*p* < 0.05). The veins diameters and lymphatic vessels output in Types IA, IB, IIA and IIB were more dilated (*p* < 0.05).

**Conclusions:**

This research demonstrated that Type IA, IB, IIA and IIB venous LNFs can retrogradely receive venous blood supply; they can survive, produce a lymphatic recanalization and integrate with the surrounding tissue, despite lymph node structural changes. Our results will improve the understanding of the survival mechanism of venous LNFs and will help researchers to design new studies or lymphatic models and eventually find an alternative procedure for the surgical treatment of lymphedema.

Highlights

- Venous lymph node flaps (LNF) were divided into five types.
- The venous lymph nodes structures were different from arteriovenous lymph nodes.
- Types IA, IB, IIA and IIB showed better clinical application potentials.

## Background

A major contribution to medicine regarding the circulatory system was provided by a Greek physician who lived between 129 and c. 200/c. 216 AD in the Roman Empire [1]. He was the first to describe the circulatory system as consisting of two separate one-way systems of distribution. His theory on the circulatory system remained unchallenged until 1628 when William Harvey significantly outlined the motion of the heart and the circulation of blood throughout the body [2]. Understanding how the blood moved in a constant circle, how the peripheral venous valves operated and how the arterial and venous blood were diverse yet interconnected with each other were all fundamentally described by Harvey. Nowadays, his concepts are still valid with research expanding on his theories. Vascular tissue perfusion can be provided by alternative surgical methods where the blood flow occurs in a different or altered direction than normal [3]. Nonconventional vascular perfusion can be created bypassing the capillary circulation using arterialized venous, total arterial or total venous flaps or by retrograde ‘reverse-flow’. A physiological explanation of the mechanism behind the function of nonconventional vascular flaps is provided by the work of Duling and Berne, which showed that in normal tissue ~50% of the oxygen is extracted from the circulation before blood reaches the capillaries [4]. Based on these findings, in the past few decades plastic and reconstructive surgeons have described several experimental and clinical studies of free-tissue transfers using a vein instead of an artery [5,6]. There are mainly two types of venous flap. The ‘arterialized venous flap’ that is nourished by arterial blood through the venous system of the flap and the ‘pure’ venous flap, described by Honda *et al.*, that is nourished by venous blood through the venous system of the flap. The ‘pure’ venous flap does not require sacrificing arteries at the donor or recipient site in contrast to the arterialized venous flap [7–9].

With the development of reconstructive microsurgery and technological advances, as well as a better understanding of the micro-anatomy, today microsurgical reconstructive techniques can be applied to different fields such as perforator flaps, complex digital replantations, lymphatic anastomosis, etc. [10–12]. Among the innovative applications of microsurgery has been the advancement of lymphatic reconstruction for the treatment of lymphedema [12–14]. In particular, promising and consistent results have been shown by transferring vascularized lymph nodes to the affected limb [15,16].

Although vascularized lymph node transfer (VLNT) is generally considered a safe procedure [17], there are still a few concerns regarding the difficulties in the surgical dissection and the management of complications. Subsequently, the authors have described and classified alternative vascular constructs using venous flow-through lymph node flaps (LNFs) and arterialized venous flow-through LNFs [18]. These novel microsurgical techniques can offer several advantages: firstly an easier dissection during flap harvesting, and in addition, acting as an adjuvant technique in case of a thrombosed or damaged artery within the LNF or in case the main vessels are accidentally separated from the flap. The venous or arterialized LNF offers an opportunity to be separated into more than two parts and can then be inserted in various manners depending on the surgical circumstances [19].

The purpose of this study was to describe and explore different constructs of VLNT based on venous blood flow without an arterial inflow in a mouse model, evaluate their blood flow microcirculation through indocyanine green (ICG) angiography, and investigate the lymphatic drainage function and the lymph nodes’ structures. The results would help to better understand the survival mechanism of venous LNFs and to ultimately explore different applications of alternative VLNT for lymphedema treatment.

## Methods

### Animals

After obtaining authorization from the Institution of the Animal Care Committee, 48 female Sprague–Dawley (SD) rats with an average weight of 220 g (ranging from 210–230 g), at 8 weeks of age, were used for the study. The rats were managed in accordance with the institutional animal research guidelines.

### Study design and surgical technique

**Figure 1:**
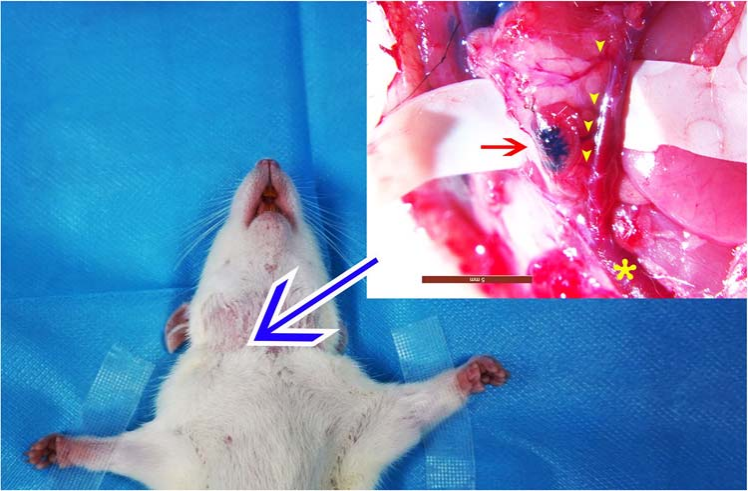
The donor sites of venous lymph node flaps. Blue arrow, the neck area; red arrow, lymph node highlighted by methylene blue; star, external jugular vein; yellow arrow heads, branches of the anterior branch of the external jugular vein

Experiments were conducted by dividing the SD rats into six groups, each containing eight SD rats (five experimental groups using different types of venous LNFs and a control group using arteriovenous LNFs). The cervical LNF [20] was used as a donor site for the venous LNF (Figure 1).

Rats were anesthetized by intraperitoneal injection of 10% chloral hydrate solution and put in the supine position.

Before surgery, 0.15 ml of methylene blue (10 mg/ml) was intracutaneously injected into the right neck, which was close to the horizontal line of the lower lip. After a 20 min interval, incisions were performed on the skin in order to locate the anterior and posterior branches of the external jugular vein, as well as the associated lymphatic and adipose tissues that had been stained with methylene blue and were nourished by the anterior branch of the external jugular vein. The LNF was detached from the surrounding tissues and the artery supplying the LNF was ligated and cut.

**Figure 2:**
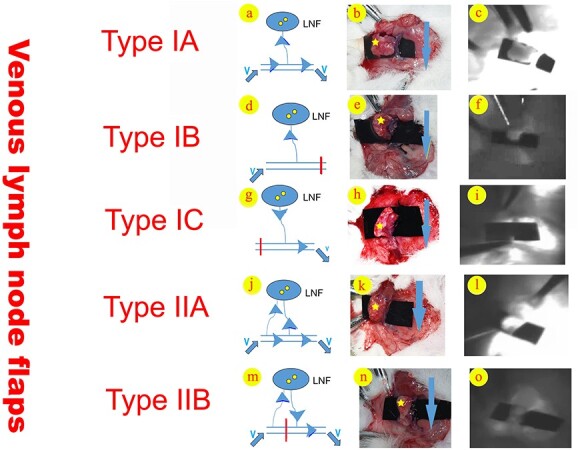
Types of venous lymph node flaps. (**a**) Diagram of Type IA; (**b**) Type IA in SD rats; (**c**) indocyanine green angiography for Type IA; (**d**) diagram of Type IB; (**e)** Type IB in SD rats; (**f**) indocyanine green angiography for Type IB; (**g**) diagram of Type IC; (**h**) Type IC in SD rats; (**i**) indocyanine green angiography for Type IC; (**j**) diagram of Type IIA; (**k**) Type IIA in SD rats; (**l**) indocyanine green angiography for Type IIA; (**m**) diagram of Type IIB; (**n**) Type IIB in SD rats; (**o**) indocyanine green angiography for Type IIB. Blue arrows, direction of venous blood flow; red vertical lines, the position of ligation and cutting; star, LNF. *LNF* Lymph node flap, *V* venous blood

Based on the number of veins nourishing the flap, we divided the venous LNFs into two main groups: single-vein-supplied LNF (Type I) and multi-veins-supplied LNF (Type II). The first group includes three subtypes: the venous branch supplied (Type IA), the terminal end of venous reflux supplied (Type IB) and lymph nodes located at the beginning of venous reflux (Type IC). The second group includes two subtypes: the venous branch supplied (Type IIA) and the flow-through (Type IIB) (Figure 2). The control group was composed of arteriovenous LNFs without any surgical intervention. The thickness of all the flaps is 0.95 (0.95–0.97) mm.

### Assessment of venous LNF perfusion

After the successful construction of the venous LNF, a light absorbing material was placed under the LNF to isolate the fluorescence interference from the underlying tissue so as to improve accuracy (Figure 2). Then, ICG solution (1 mg/100 g of rat) was injected into the femoral vein. Angiography was performed while monitoring with a near-infrared probe (Photodynamic Eye, Hamamatsu Photonics KK, Hamamatsu, Japan). The blood supply of each venous LNF type was evaluated using ICG angiography.

### Evaluation of lymphatic connection restoration with surrounding tissue

After the construction of the venous LNF, the wound of the neck was sutured and SD rats were given oral antibiotics for 3–4 days. At week 7 post-treatment, the SD rats were anesthetized for intracutaneous injection of methylene blue for identification of the venous LNF prepared 7 weeks earlier. The internal lymph nodes were examined for methylene blue uptake.

### Histological assessment

Seven weeks later, the lymph node model was re-explored and the lymph nodes were detached. Subsequently, 5 μm thick sections were obtained and stained with hematoxylin and eosin (HE). Three sections of each lymph node were chosen randomly and observed using optical microscopy by two pathologists to evaluate lymph node structures, with findings recorded as either ‘√’ for present or ‘X’ for absent. Simultaneously, IMAGE J software (National Institutes of Health NIH, Bethesda, MD, USA) was used to analyze and measure the tubular structure in the lymph node images. The differences in artery diameter, vein diameter, afferent and efferent lymphatics diameter, high endothelial venules diameter, and the number of cells between the ‘venous LNF’ and control group ‘arteriovenous LNF’ were measured and recorded.

### Ethics approval and consent

The Ethics Commission approved research protocols for the Use of Experimental Animals at the Jiao Tong University-Ninth People’s Hospital of Shanghai. The study protocol was agreed by the local ethics committee of the Jiao Tong University-Ninth People’s Hospital of Shanghai.

### Statistical methods

The data for arteries, veins, afferent lymphatics, efferent lymphatics, high endothelial venules and ratio of cells in each lymph node group obey normal distribution, respectively. One-way analysis of variance (ANOVA) and the *post hoc* Tukey test were conducted for inter-group and pairwise comparison. All data analyses were conducted using SPSS version 19.0 (IBM SPSS Inc., New York, NY, USA); significance *p* < 0.05.

## Results

### Construction of the venous LNFs animal model

Based on previous studies and according to our hypothesis, five types of venous LNFs were successfully created in SD rats (Figure 2).

### Intraoperative evaluation of the blood supply of the venous LNFs

After the construction of several venous LNF types, ICG was injected into the rat’s femoral vein. One minute later, ICG could be seen flowing into the Types IA, IB, IIA and IIB venous LNFs, as shown in Figure 2, and Videos 1, 2, 3 and 4, see online supplementary material. These four venous LNF types were shown to retrogradely obtain blood supply via the vein branch, which demonstrated a higher flap survival rate. In contrast, the Type IC group was not developed, suggesting poor flap survival (Figure 2, and Video 5, see online supplementary material). The control group (arteriovenous LNF) obtains blood supply via artery (Video 6, see online supplementary material).

### Evaluation of lymphatic connection and integration with the surrounding tissue

**Figure 3:**
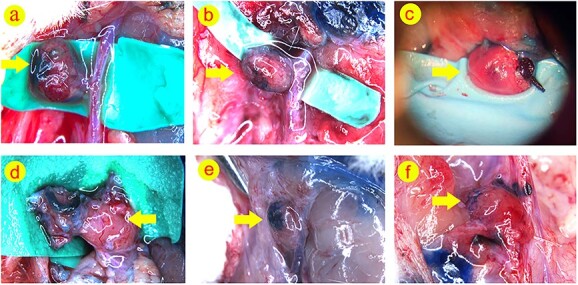
Lymph nodes highlighted by methylene blue 7 weeks after surgery. (**a**) Methylene blue could be seen in the lymph node of arteriovenous group; (**b**) methylene blue could be seen in the lymph node of Type IA group; (**c**) ethylene blue could be seen in the lymph node of Type IB group; (**d**) no obvious methylene blue could be seen in the lymph node of Type IC group; (**e**) methylene blue could be seen in the lymph node of Type IIA group; (**f**) methylene blue could be seen in the lymph node of Type IIB group. Arrow, lymph node area

Results showed that intracutaneously injected methylene blue had the ability to traverse through the lymph nodes in the arteriovenous lymph nodes group. Notably, at week 7 post-operation, methylene blue was detected in the arteriovenous lymph nodes group and the four venous LNF types (Types IA, IB, IIA and IIB) (Figure 3). These findings highlight that venous LNFs possess the capacity to produce lymphatic recanalization with the surrounding tissue and that the internal lymph nodes allow for the collection of lymphatic fluid. In contrast, the lymphatic drain function of the Type IC venous LNF was significantly reduced or absent (Figure 3).

### Lymph node structural analysis

**Figure 4:**
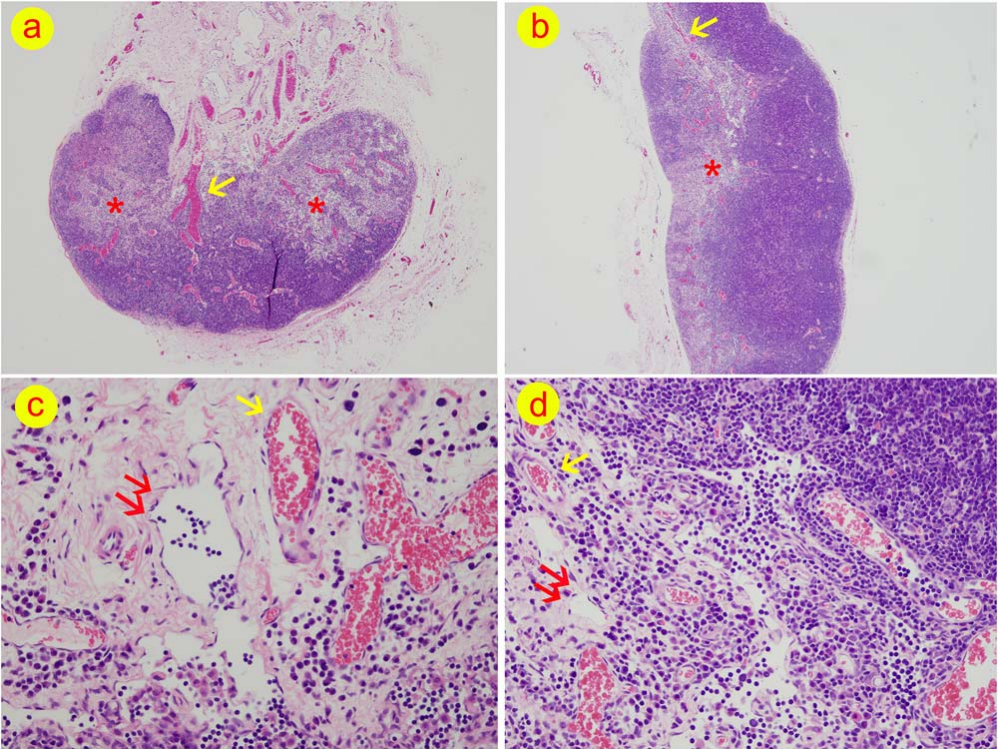
The structure of lymph nodes from venous lymph node flaps (LNFs). (**a**) Hematoxylin–eosin-stained pictures of lymph nodes from venous LNFs (40x). Medullary sinus distributes diffusely and it occupies the major component of the node. The amount of cells in lymph nodes are reduced in comparison with the control groups. At the hilum of the node, thick veins were identified. (**b**) Hematoxylin–eosin-stained pictures of lymph nodes from arteriovenous lymph node groups (40x). Medullary sinus was seen in the central portion of the node. At the hilum of the node, the diameters of veins were small. (**c**) The hilum of lymph nodes from venous lymph node flaps (400x). Dilated efferent lymphatic vessels and veins were discovered in all types of venous lymph nodes except Type IC. (**d**) The hilum of lymph nodes from arteriovenous lymph node groups (400x). Star, the area of medullary sinus; double arrow, efferent lymphatic vessels; single arrow, veins

At week 7 following the surgical procedure, the cervical lymph nodes of the venous lymphatic group and arteriovenous lymph nodes group were extracted and subjected to HE staining. Notably, upon comparison of the interstitial component, the distribution of the medullary sinus in the arteriovenous lymph nodes was observed to be primarily concentrated in the central region. In contrast, the medullary sinus in the venous lymph nodes was found to be diffusely distributed (Figure 4). However, no significant differences were noted in the remaining interstitial structure of the venous lymph nodes when compared to the arteriovenous group (Table 1).

**Table 1:** Histologic features of normal cervical node of rat and cervical nodes of venous lymph node flaps

**Component of lymph node**	**Control**	**Type IA**	**Type IB**	**Type IC**	**Type IIA**	**Type IIB**
**Lymphatic/sinus component (interstitial component)**						
Capsule	√	√	√	√	√	√
Trabeculae	√	√	√	√	√	√
Subcapsular sinus	√	√	√	√	√	√
Medullary sinus	√ (In the central portion of the node)	√ (Major component of the node)	√ (Major component of the node)	√ (Major component of the node)	√ (Major component of the node)	√ (Major component of the node)
Sinus histiocytosis	√	√	√	√	√	√
Fine structural skeleton of fibroblastic reticular cells and reticulin	√	√	√	√	√	√
**Stromal component**						
Cortex (B-cells)	√	√	√	√	√	√
Paracortex (T-cells)	√	√	√	√	√	√
Primary follicles	√	√	√	√	√	√
Secondary follicles	√	√	√	√	√	√
Medullary cords	√	√	√	√	√	√
**Vascular component**						
High endothelial venules	√	√	√	√	√	√
Artery–vein	√	√ (Narrow artery, dilated veins)	√ (Narrow artery, dilated veins)	√	√ (Narrow artery, dilated veins)	√ (Narrow artery, dilated veins)
Afferent and efferent lymphatics	√	√ (Dilated efferent lymphatics)	√ (Dilated efferent lymphatics)	√	√ (Dilated efferent lymphatics)	√ (Dilated efferent lymphatics)

√ Present

Furthermore, in the stromal components, cortex, paracortex, primary follicles, secondary follicles, and medullary cords were identified in all six lymph node groups (Table 1). The inter-group difference in the proportion of cells was found to be significant (*p* < 0.001). The proportion of cells in each venous lymph node group was significantly reduced when compared to the arteriovenous lymph node group (*p* < 0.05), indicating a reduction in the matrix components (Table 2).

**Table 2:** The comparison of cell proportion and vascular components in the experimental groups

**Groups**	**Cell proportion (mean ± SD) (%)**	**Artery diameter (mean ± SD) (μm)**	**Vein diameter (mean ± SD) (μm)**	**Efferent lymphatic vessel diameter (mean ± SD) (μm)**	**Afferent lymphatic vessel diameter (mean ± SD) (μm)**	**High endothelial venules diameter (mean ± SD) (μm)**
Arteriovenous (control)	72.158 ± 1.966	69.419 ± 7.968	23.171 ± 6.445	24.314 ± 3.365	30.913 ± 1.592	21.150 ± 3.001
Type IA	54.823 ± 1.995	31.409 ± 2.709	137.720 ± 9.339	142.984 ± 14.087	31.424 ± 1.707	21.852 ± 1.961
Type IB	60.645 ± 2.581	25.643 ± 2.912	146.552 ± 13.951	155.384 ± 18.719	31.783 ± 1.780	21.426 ± 2.358
Type IC	64.276 ± 2.393	29.943 ± 2.736	29.216 ± 6.761	26.100 ± 4.079	31.790 ± 2.100	22.224 ± 2.498
Type IIA	54.293 ± 8.404	27.548 ± 7.195	141.092 ± 13.882	148.067 ± 14.293	30.987 ± 2.128	21.400 ± 2.501
Type IIB	55.759 ± 6.969	25.473 ± 3.707	149.592 ± 14.935	152.603 ± 13.215	32.274 ± 1.552	22.932 ± 2.562
Type IA *vs* control	*p* < 0.001	*p* < 0.001	*p* = 0.010	*p* < 0.001	*p* = 0.993	*p* = 0.993
Type IB *vs* control	*p* < 0.001	*p* < 0.001	*p* < 0.001	*p* < 0.001	*p* = 0.930	*p* = 1.000
Type IC *vs* control	*p* = 0.025	*p* < 0.001	*p* = 0.895	*p* = 0.999	*p* = 0.927	*p* = 0.954
Type IIA *vs* control	*p* < 0.001	*p* < 0.001	*p* < 0.001	*p* < 0.001	*p* = 1.000	*p* = 1.000
Type IIB *vs* control	*p* < 0.001	*p* < 0.001	*p* < 0.001	*p*< 0.001	*p* = 0.671	*p* = 0.712

Regarding the vascular component, significant inter-group differences were found in the diameter of arteries, veins and efferent lymphatic vessels (*p* < 0.001). Specifically, the diameter of arteries within the lymph node hilum was significantly smaller in all venous lymph node groups compared to the arteriovenous lymph node group (*p* < 0.05). In contrast, the diameter of veins in the lymph node hilum was significantly larger in all venous lymph node groups compared to the arteriovenous group (*p* < 0.05), with the exception of the Type IC group (*p* = 0.895). Moreover, the efferent lymphatic vessel diameter in all venous groups was substantially larger (*p* < 0.05) than that in the arteriovenous group (Figure 4), except for the Type IC group (*p* = 0.999). Conversely, no significant inter-group differences were detected in the diameter of afferent lymphatic vessels and high endothelial venules, with *p*-values of 0.656 and 0.731, respectively (Table 2).

## Discussion

The concept of the venous flap was first introduced in 1981 by Nakayama *et al*. [21] who referred to a flap perfusion that solely relied on the venous system. Three methods of flap perfusion were described: (1) arteriovenous shunt (i.e. reverse blood flow from a venous system to an arterial system via arteriovenous traffic branches); (2) countercurrent (i.e. through intermittent blood flow entering the capillary along the vein and then flowing out the vein); and (3) capillary bypass (i.e. blood flowing into the venous system until the angiogenesis enters the arterial system. Subsequently, Chen *et al*. described the classification of venous flaps in more detail [22]. Although the exact blood flow pattern of the venous flap was not adequately investigated, clinical practice showed that the venous flap could survive and work; therefore, it can be used as an alternative procedure in case of an unreliable arterial blood supply of the flap [23].

After administration of ICG to the venous LNFs, we found that the Type IC venous LNF could not obtain the reverse venous blood supply. However, Types IA, IB, IIA and IIB venous LNFs received a sufficient blood supply. It is established that the promotion of venous return comes from the difference in the blood pressure between both vessel ends [5]. When the venous LNF loses the arterial supply, the microcirculation fluid pressure in the flap gradually decreases. In addition, when the pressure is lower than the fluid pressure in the primary vein, the flow will retrogradly flow along the vein into the flap [24]. However, we think that the poor perfusion of the Type IC venous LNF resulted from the (upper) primary venous vein cutoff. Therefore, the flap needs a retrograde supply from the secondary vein, with a higher pressure difference. In addition, the pressure of the original fluid in Type IC flaps is much higher than in the primary vein. Thus, temporarily, it is not possible to provide enough reverse power, causing a negative angiography result. Later, when the pressure in the flap is reduced to a certain gradient, retrograde blood might progressively flow into the flap [9].

The mouse model is widely adopted for microsurgical research in lymphatic surgery and it has contributed to the improvement of several innovative surgical procedures such as VLNT [25].

However, some important anatomical and physiological differences between humans and rats need to be considered. In the rat, a lymphatic vessel sometimes converges with other lymphatic vessels within a lymphosome, but connected to a single sentinel node only [26].

In the human body, most of the peripheral lymph nodes are distributed along the primary superficial or deep veins. The peripheral lymph nodes, the surrounding lymphatic vessels and their veins form a functional lymphatic unit. In addition, lymph can return to the lymph nodes through the lymphatic unit and be pumped into the venous systems [27]. The structure of the lymph nodes includes traffic branches connecting internal arteries and veins, where materials interchange between lymph nodes and the vascular system [28,29]. Lymph nodes can secrete cytokines such as VEGF-C, promoting lymphangiogenesis near the lymph nodes, increasing local lymphatic vessel density, and stimulating the recanalization of lymphatic vessels with lymph nodes and recipient lymph nodes [30]. Cheng *et al*. [31] described the lymph nodes’ pump function as collecting lymphocytes from surrounding tissues and pumping them into the venous system. It was also demonstrated that the transplanted lymph nodes could recover their lymph collection functions within 45 days of surgery [32]. Further research showed that subcutaneous lymphatic system regeneration started 4 days after the surgery, with the integration of the recipient tissue taking place 5 days postoperatively [33,34].

In this experimental model, the Types IA, IB, IIA and IIB venous LNFs showed positive results after lymph node angiography and intradermal injection of methylene blue 7 weeks after the transplantation, whilst the Type IC venous LNFs were negative. This indicated the survival of lymph nodes in the Types IA, IB, IIA and IIB venous LNFs and the restoration of lymph drainage functions from nearby tissues whilst Type IC venous lymph nodes failed.

A capsule covers the lymph node’s surface, and the connective tissue in the hilum extends into the lymph node, forming numerous trabeculae. The parenchyma is divided into the surrounding cortex and central medulla. The two structures have no clear boundaries and are interconnected via the lymphatic sinus, penetrating the cortex and medulla [35]. *Wheater’s Functional Histology* textbook described the three functional regions of lymph nodes: the lymphatic and sinus component, the matrix, and the vascular region [36]. The stromal area is the area of the lymph nodes filled with dense lymphocytes, primarily responsible for the immune response of lymph nodes. The vascular area provides the lymphocytes with a route to the lymph nodes and the substances required for metabolism. Lastly, the lymphatic and sinus area are the interstitial part of the lymph nodes responsible for lymph flow from the afferent lymphatics to the efferent lymphatics. It is also involved in the formation of the lymphoid skeleton.

Interestingly, the lymphatic and sinus area can be described as being similar to a sponge that has several open pores composed of reticular fibers and fibroblasts. Thus, when lymph fluid enters the subcapsular lymphatic sinus through the afferent lymphatics, it passes through several circular sinus structures with a small diameter, eventually reaching the efferent lymphatics. The area provides a higher resistance to lymph flow, which also provides the ability of the lymph to filter antigens, the function of the immune response [32].

The present study found that the structure of the lymph nodes in the venous LNF was typical, the medullary sinus was diffusely distributed and the matrix component was reduced. Studies have shown that 90% of lymphocytes in typical lymph nodes were brought by arterial blood flow, while 10% were from the afferent lymphatic vessels [36,37]. The lymph nodes in the present study were retrogradely supplied by the venous blood flow. Assuming that the venous blood pressure is lower than that of the arteries, the blood volume entering the lymph nodes via the venous system is less than the arterial blood supply per unit time. Consequently, it resulted in less lymphocyte content than typical lymph nodes. Moreover, the sinus could not be filled, and HE staining showed that the medullary sinus was diffusely distributed and the matrix component was reduced.

In the present study, the veins and efferent lymphatic vessels from Type IA, IB, IIA and IIB venous LNFs were dilated, and the artery diameter was reduced. The result could be explained by: (1) the lymphocyte count in the lymph nodes being reduced; (2) the medullary sinus was diffusely distributed, resulting in an increase in lymph node pores and a reduction in fluid resistance [32]; and (3) the lymphatic reflux increased lymphatic flow, causing the efferent lymphatic vessels to be dilated. In addition, there were traffic branches and substance exchange between the lymphatic vessels and the veins in the lymph nodes that may cause vein dilation. Furthermore, when the blood of the venous lymph nodes is fed through the vein, the artery cannot be filled and the artery’s diameter becomes smaller.

Although this investigation has demonstrated that the venous LNF can adequately retrieve the lymph fluid from the recipient area, its efficacy to treat lymphedema and to be translated to the clinical setting needs to be confirmed by further studies.

## Conclusions

This research showed that Types IA, IB, IIA and IIB venous LNFs retrogradely received a venous blood supply, producing a lymphatic recanalization whilst integrating with the surrounding tissue contributing to its survival, despite some modifications to the lymph node structures. Taking into consideration the anatomical differences between species and the experimental model, we suppose that Types IA, IB, IIA and IIB venous LNFs have the potential to treat lymphedema. Our results may help researchers to design new studies or lymphatic models and eventually find an alternative procedure for the surgical treatment of lymphedema.

## Abbreviations

HE: Hematoxylin and oesin; ICG: Indocyanine green; LNF: Lynph node flap; SD: Sprague–Dawley; VLNT: Vascularized lymph node transfer.

## Funding

The national natural science foundation of China (Grant Number: 81772098), Clinical Multi-Disciplinary Team Research Program of 9th People’s Hospital, Shanghai Jiao Tong University School of Medicine (Grant Number: 2017–1-007), Clinical Research Program of 9th People’s Hospital, Shanghai Jiao Tong University School of Medicine (Grant Number: JYLJ027), Shanghai Municipal Education Commission Gaofeng Clinical Medicine Grant Support (Grant Number: 20152227), The national natural science foundation of China (Grant Number: 82000456) and Scientific research foundation of Shanghai Municipal Commission of Health and Family Planning (Grant Number: 20154Y0023).

## Authors’ contributions

KL, CC and FN contributed to data collection, data analysis and writing; YW, SF, WX and NFL contributed to data collection; PM, KL and YZ contributed to study design and writing.

## Ethics approval and consent to participate

The Ethics Commission approved research protocols for the use of experimental animals at the Jiao Tong University-Ninth People’s Hospital of Shanghai. The study protocol was agreed by the local ethics committee of the Jiao Tong University-Ninth People’s Hospital of Shanghai.

## Conflict of interests

There is no conflict of interest for all the authors.

## Data availability statement

All data or related information supporting the conclusions of the review is included in the article.

## Supplementary Material

video_1_tkad019

video_2_tkad019

video_3_tkad019

video_4_tkad019

video_5_tkad019

Video_6_tkad019

Supplementary_Video_Legend_tkad019
