# Supplementary material for: Vascularized lymph node flaps can survive on venous blood without an arterial inflow: an experimental model describing the dynamics of venous flow using indocyanine green angiography (With video)
Source: Burns Trauma. 2023 Jul 19;11:tkad019. doi: 10.1093/burnst/tkad019 (PMC10355992; doi:10.1093/burnst/tkad019)
Supplement: Supplementary_Video_Legend_tkad019 [file supplementary_video_legend_tkad019.docx]

Video 1. Indocyanine green angiography for TYPE IA.

Video 2. Indocyanine green angiography for TYPE IB.

Video 3. Indocyanine green angiography for TYPE IIA.

Video 4. Indocyanine green angiography for TYPE IIB.

Video 5. Indocyanine green angiography for TYPE IC.

Video 6. Indocyanine green angiography for control group （arteriovenous lymph node flap）.
